# Bi_2_Te_3_/Carbon Nanotube Hybrid Nanomaterials as Catalysts for Thermoelectric Hydrogen Peroxide Generation

**DOI:** 10.3390/molecules29225242

**Published:** 2024-11-06

**Authors:** Chunlei Li, Shun Li, Long Zhao, Jianming Zhang

**Affiliations:** School of Chemistry and Chemical Engineering, Jiangsu University, Zhenjiang 212000, China; 2222112058@stmai.ujs.edu.cn (C.L.); shun@ujs.edu.cn (S.L.); 1000004719@ujs.edu.cn (L.Z.)

**Keywords:** thermoelectric, catalysis, hydrogen peroxide, carbon nanotube, charge separation

## Abstract

Harnessing waste heat from environmental or industrial sources presents a promising approach to eco-friendly and sustainable chemical synthesis. In this study, we introduce a thermoelectrocatalytic (TECatal) system capable of utilizing even small amounts of heat for hydrogen peroxide (H_2_O_2_) production. We developed a nanohybrid structure, combining carbon nanotubes (CNTs) and Bi_2_Te_3_ nanoflakes (Bi_2_Te_3_/CNTs), through a one-pot synthesis method. Bi_2_Te_3_, as a thermoelectric (TE) material, generates charge carriers under a temperature gradient via the Seebeck effect, enabling them to participate in surface redox reactions. However, the rapid recombination of these charge carriers greatly limits the TECatal activity. In the Bi_2_Te_3_/CNTs nanohybrid system, the introduction of CNTs substantially enhances the efficiency of H_2_O_2_ production, as the strong bonding between CNTs and Bi_2_Te_3_, along with the excellent conductivity of CNTs, facilitates charge carrier separation and transport, as confirmed by TE electrochemical tests. This study underscores the significant potential of thermoelectric nanomaterials for converting waste heat into green chemical synthesis.

## 1. Introduction

In industrial production, on average, only about one-third of the primary energy is effectively utilized, with approximately 75% lost as waste heat [1,2]. In sectors such as chemical and petrochemical industries, heat plays an essential role in maintaining the necessary conditions for chemical processes and promoting reactions. This widespread presence of temperature differentials in the environment points to the significant potential for capturing waste heat or temperature gradients for eco-friendly and cost-effective synthesis [3]. Thermoelectric (TE) materials have the ability to create a steady electric field when exposed to a temperature gradient (ΔT), a phenomenon known as the Seebeck effect, which allows for the conversion of thermal energy into electrical energy [4,5,6,7,8,9,10]. Recent studies have showcased the successful application of TE materials’ Seebeck effect to act as catalysts, converting thermal energy into chemical energy—a process termed thermocatalysis (TECatal) [11,12,13,14,15]. In TE materials, the free charges generated by the thermoelectric effect interact with surrounding intermediates, forming active radicals that can drive surface redox reactions.

Bismuth telluride (Bi_2_Te_3_) is one of the most researched TE materials due to its favorable thermoelectric properties near room temperature and its cost-effectiveness [16,17,18,19], making it an ideal candidate for applications such as photothermal therapy, thermal sensors, and catalysis [20,21,22,23]. Despite these advantages, the TECatal performance of Bi_2_Te_3_ is limited by its relatively low carrier mobility [24,25,26], which hampers its catalytic efficiency. Thus, designing a more efficient Bi_2_Te_3_-based thermocatalytic system and developing innovative catalytic pathways remains an important challenge. Integrating one-dimensional carbon nanostructures, such as carbon nanotubes (CNTs) bearing excellent conductivity, could be one of the effective ways to enhance charge transport [27,28].

In this study, we developed a TECatal system for hydrogen peroxide (H_2_O_2_) production from pure water, operating under a modest temperature gradient, using a Bi_2_Te_3_-based nanohybrid catalyst. The catalyst was constructed by integrating n-type Bi_2_Te_3_ nanoflakes with multi-walled carbon nanotubes (CNTs) to form Bi_2_Te_3_/CNT nanohybrids. Under a temperature difference of 46 K, the efficiency of H_2_O_2_ production for Bi_2_Te_3_ nanoflakes is very low; by contrast, it was enhanced significantly using the Bi_2_Te_3_/CNT nanohybrids. Further thermoelectric electrochemical tests confirmed that CNTs played a critical role in enhancing the separation of Seebeck effect-induced charges owing to the excellent conductivity of the CNTs. This proof-of-concept study underscores the great potential of TECatal for promoting sustainable synthesis by utilizing waste heat energy.

## 2. Results and Discussion

**Figure 1:**
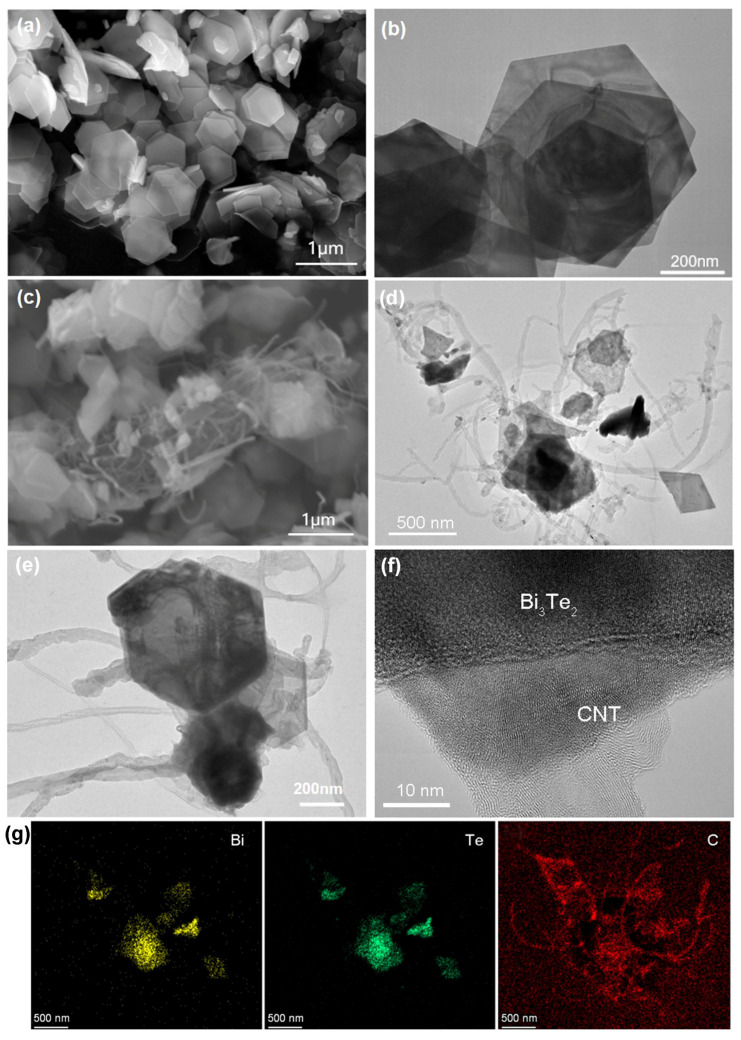
TE catalyst structural characterizations. (**a**) SEM and (**b**) TEM images of the Bi_2_Te_3_ nanoflakes. (**c**) SEM and (**d**,**e**) TEM images of the Bi_2_Te_3_/CNT heterostructures. (**f**) HR-TEM image of the Bi_2_Te_3_/CNT heterostructure. (**g**) EDS elemental mapping image of the Bi_2_Te_3_/CNT heterostructures shown in (**d**).

As schematically illustrated in Figure 1a, the Bi_2_Te_3_/CNT nanohybrids were synthesized by introducing CNTs into the Bi_2_Te_3_ precursor solution, containing Bi_2_O_3_, TeO_2_, Polyvinyl pyrrolidone (PVP), and NaOH, followed by a simple hydrothermal process. The morphology of the as-synthesized catalysts was examined using scanning electron microscopy (SEM) and transmission electron microscopy (TEM). The SEM (Figure 1a) and TEM (Figure 1b) images reveal that the Bi_2_Te_3_ nanomaterials exhibit a uniform hexagonal plate-like structure, with dimensions ranging from approximately 300 to 500 nm (corner to corner), and a thickness of several tens of nanometers. The SEM (Figure 1c) and TEM images of the Bi_2_Te_3_/CNT nanohybrids (Figure 1d,e) demonstrate that the hexagonal Bi_2_Te_3_ nanoplates are successfully integrated with wire-like CNTs, confirming their successful hybridization during the one-pot synthesis method of Bi_2_Te_3_ in the presence of CNTs in the precursor solution. The high-resolution TEM image of the Bi_2_Te_3_/CNT nanohybrid interface in Figure 1f clearly shows the typical multiwall structure of the CNTs. The energy dispersive X-ray spectroscopy (EDS) elemental mapping image in Figure 1g displays that the key elements of Bi, Te, and C are distributed following their TEM observation, again confirming the successful construction of Bi_2_Te_3_/CNT nanohybrids.

**Figure 2:**
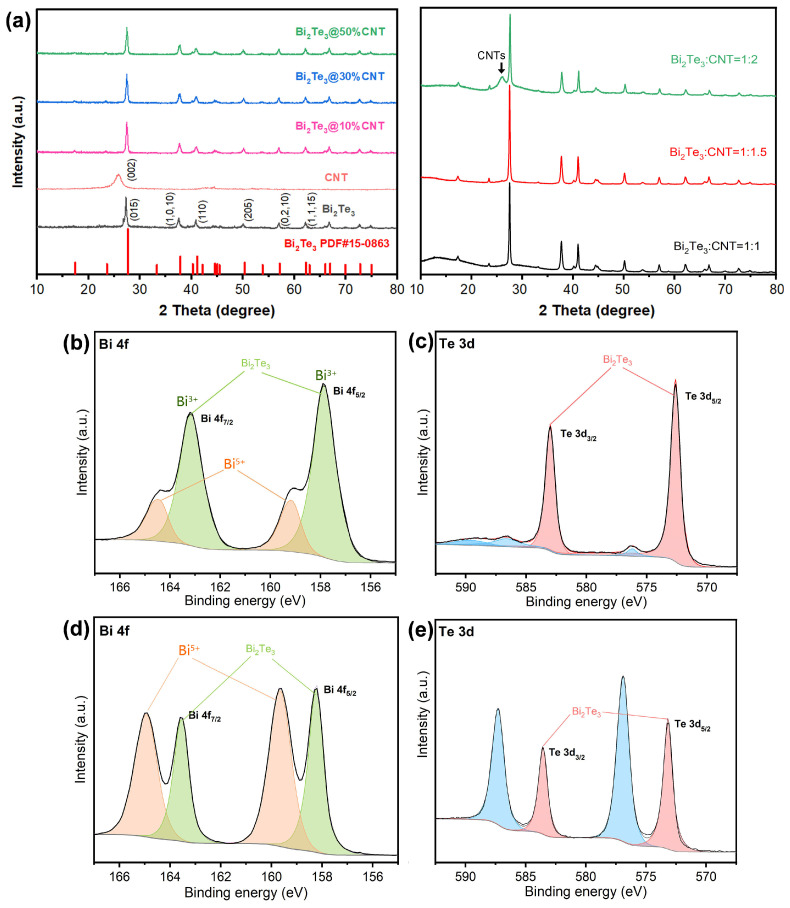
(**a**) Left: XRD patterns of Bi_2_Te_3_, CNTs and Bi_2_Te_3_/CNT (CNT content = 10%, 30%, 50%); Right: XRD patterns of Bi_2_Te_3_/CNTs with higher CNT content. (**b**) Bi 4f and (**c**) Te 3d XPS spectra of Bi_2_Te_3_. (**d**) Bi 4f and (**e**) Te 3d XPS spectra of Bi_2_Te_3_/CNT.

The crystal structures of both the Bi_2_Te_3_ nanoplates and the Bi_2_Te_3_/CNT nanohybrids with varying CNT contents were analyzed using X-ray diffraction (XRD), as shown in Figure 2a-left. The XRD patterns for all samples are consistent, displaying six prominent peaks that correspond to the (0 1 5), (1, 0, 10), (1 1 0), (2 0 5), (0, 2, 10), and (1, 1, 15) planes of the rhombohedral Bi_2_Te_3_ lattice (JCPDS card no. 15-0863). Notably, no distinct peaks corresponding to CNTs were observed in the spectra of the nanohybrids, even for samples with a high CNT content (approximately 50% in mass ratio). This absence could be due to the fact that the week signals of CNTs are submerged by the strong diffractions of Bi_2_Te_3_ crystals. To confirm this inference, additional samples with a much higher CNT content were analyzed using XRD. As illustrated in Figure 2a-right, only for the sample with a CNT content as high as 200%, a small band corresponding to the (002) diffraction of CNTs can be detected (marked with black arrow).

The chemical states of the primary elements of the catalyst were examined using X-ray photoelectron spectroscopy (XPS). As shown in Figure 2b,c, both the Bi 4f and Te 3d spectra for the Bi_2_Te_3_ nanoplates display two distinct peaks, accompanied by small shoulder bands on the higher binding energy side. Interestingly, the incorporation of CNTs into Bi_2_Te_3_ to form the Bi_2_Te_3_/CNT nanohybrids resulted in a noticeable change in the spectrum. In Figure 2d,e, the shoulder bands at the higher binding energy sides of both Bi 4f and Te 3d become remarkably prominent, which can be attributed to a great surface charge redistribution caused by the junction between Bi_2_Te_3_ and CNTs. This observation suggests a strong interaction or bonding between the Bi_2_Te_3_ and CNTs.

**Figure 3:**
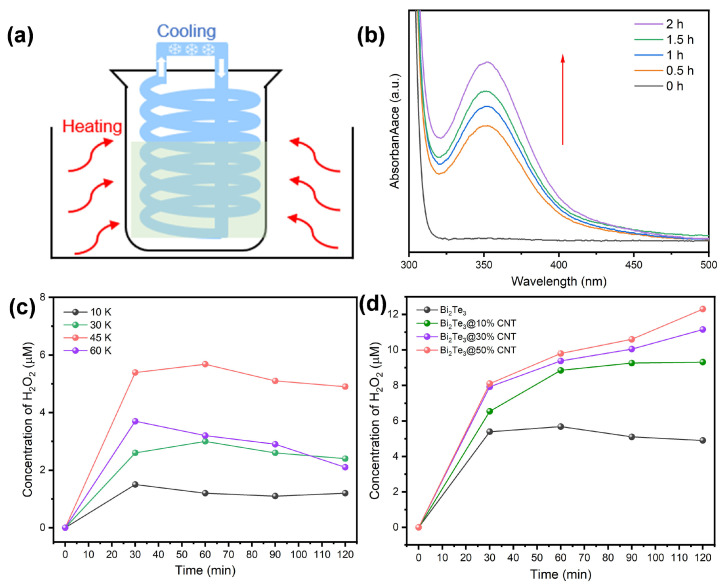
(**a**) Schematic illustration of TECatal experimental setup. (**b**) Colorimetric UV–Visible absorption spectral evolution of catalytically generated H_2_O_2_ using Bi_2_Te_3_/50% CNT as a catalyst. (**c**) Catalytic reaction efficiency diagram of Bi_2_Te_3_ at a temperature difference of 10 K, 30 K, 45 K, and 60 K. (**d**) Catalytic reaction efficiency diagram of Bi_2_Te_3_, Bi_2_Te_3_/10%CNT, Bi_2_Te_3_/30%CNT, and Bi_2_Te_3_/50% CNT at ΔT = 45 K.

The TECatal activity of the Bi_2_Te_3_ nanoplates and Bi_2_Te_3_/CNT nanohybrids was evaluated for hydrogen peroxide (H_2_O_2_) production. For the TECatal experiment, a suspension of the catalyst in a beaker was heated in an oil bath at varying temperatures, while a temperature gradient across the system was established by placing a cooling coil tube (cooling water temperature was set at 5 °C) into the reaction solution, as illustrated in Figure 3a. The ΔT is approximately considered as the temperature difference between the oil bath and cooling water (5 °C), which is tunable by simply changing the oil temperature. The amount of H_2_O_2_ formed during the reaction was quantified by separating the nanomaterials from the solution and measuring the UV-Vis absorbance at 351 nm (Abs 351 nm) using the conventional colorimetric method (see Section 3.6). As shown in Figure 3b, the Abs351 nm peak gradually increased over time, indicating the continuous generation of H_2_O_2_ during the reaction. The results of the temperature gradient-dependent measurements (Figure 3c) of the Bi_2_Te_3_ system revealed that increasing the ΔT from 10 K to 45 K enhanced H_2_O_2_ production from ~1.2 μM to ~5 μM in 2 h. However, when ΔT was further raised to 60 K with an oil bath temperature of 65 °C, a significant decrease in reaction efficiency was observed, likely due to the instability of H_2_O_2_ at higher temperatures [29].

At a temperature gradient (ΔT) of 45 K, the initial use of Bi_2_Te_3_ as a catalyst results in a peak H_2_O_2_ production of ~5 μM after 2 h, with the reaction largely stalling after the first hour. In sharp contrast, the Bi_2_Te_3_/CNT nanohybrid demonstrates sustained H_2_O_2_ production throughout the reaction period, achieving a final concentration of 12.3 μM within the same 2-h window (Figure 3d). As the CNT content increases from 10% to 50%, denoted as Bi_2_Te_3_/*x*CNT (*x* = 0–50% in mass ratio), a continuous improvement in reaction efficiency is observed. However, further increasing the CNT content to 70% does not lead to additional gains in catalytic performance. We thus used the Bi_2_Te_3_/50%CNT nanohybrid as the standard sample for further investigation and named it Bi_2_Te_3_/CNT. A reaction carried out using a simple mixture of Bi_2_Te_3_ and CNTs produces a yield of H_2_O_2_ similar to that obtained using Bi_2_Te_3_ nanoplates alone, suggesting that the interaction or bonding between Bi_2_Te_3_ and CNTs in the Bi_2_Te_3_/CNT nanohybrid is critical for enhancing the reaction efficiency.

**Figure 4:**
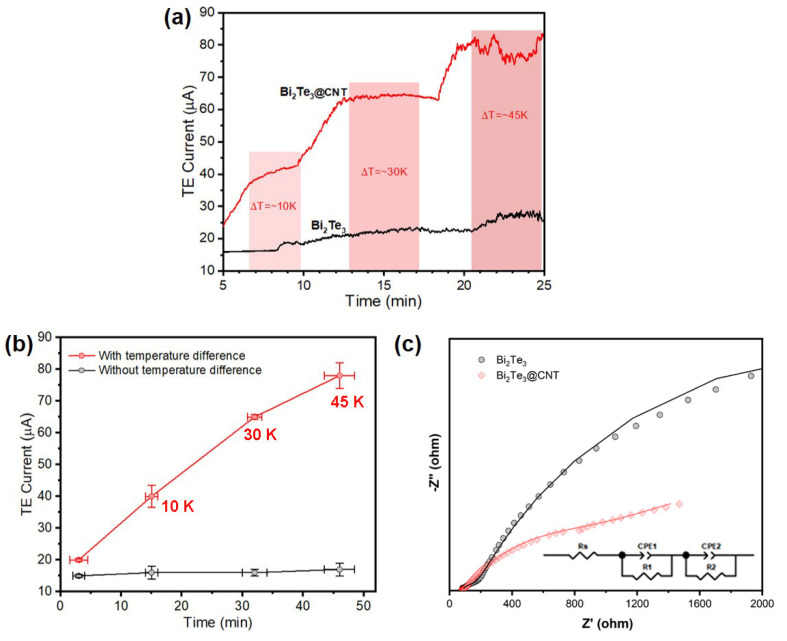
(**a**) Electrochemical TE current results with continuous temperature increase in Bi_2_Te_3_ and Bi_2_Te_3_/CNT electrodes. (**b**) TE current of Bi_2_Te_3_/CNT nanohybrids with and without temperature difference. (**c**) EIS Nyquist plots of Bi_2_Te_3_ and Bi_2_Te_3_/CNT electrodes (ΔT = 45 K).

To further evaluate the TE charge separation performance, a TE current test was performed using an electrochemical cell, with the catalysts serving as the working electrode (see Section 3.7). As shown in Figure 4a,b, increasing ΔT results in a much stronger response for the Bi_2_Te_3_/CNT nanohybrids (CNT content = 50%) compared to the pristine Bi_2_Te_3_ across a wide ΔT range. The Bi_2_Te_3_/CNT nanohybrids exhibit a continuously amplified current intensity, and the TE current remains pretty stable over time, without noticeable decay (Figure 4a), suggesting superior charge separation properties. Consistent with the catalytic tests, no TE current was detected under uniform heating for Bi_2_Te_3_/CNT nanohybrids (ΔT = 0), as presented in Figure 4b, further ruling out the possibility of thermal catalysis in this study. Figure 4c displays the Nyquist plots obtained from electrochemical impedance spectroscopy (EIS). At ΔT = 45 K, the Bi_2_Te_3_/CNT nanohybrids exhibit both significantly smaller semicircle diameters of R1 (Z′ < 200 ohm) and R2 (Z′ > 200 ohm) of the fitted EIS curve, corresponding to the interfacial and bulk resistances, respectively, compared to the Bi_2_Te_3_ nanoplates, providing further confirmation of the enhanced charge separation performance in the Bi_2_Te_3_/CNT system.

**Figure 5:**
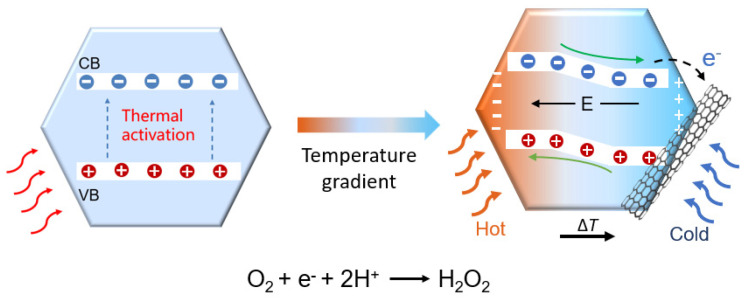
Scheme of TECatal mechanism for temperature gradient (ΔT)-induced charge separation and surface reaction processes.

In principle, under thermal equilibrium, while the free charges in Bi_2_Te_3_ can be thermally excited (Figure 5, left), they are likely to undergo rapid recombination or depletion before participating in surface reactions, leading to reduced catalytic efficiency. In contrast, upon applying a temperature gradient, the Seebeck effect causes electrons to migrate from the hot side to the cold side, creating an internal electric field across the Bi_2_Te_3_ material between its hot and cold ends (Figure 5, right). This TE electric field results in band tilting, where the band energy decreases on the positive potential side and increases on the negative potential side. This modification of the band structure improves the separation of free electron–hole pairs, enabling them to participate in surface reactions for H_2_O_2_ production. Although charge recombination still occurs, the strong interaction between CNTs and Bi_2_Te_3_ enhances charge separation, as the excellent conductivity of CNTs allows them to act as efficient electron transporters. These separated charges then contribute to the redox reactions, increasing the rate of H_2_O_2_ formation.

## 3. Materials and Methods

### 3.1. Materials

Bi_2_O_3_, TeO_2_, ZrCl_4_, KI, 2-Aminoterephthalic Acid, CNTs, and 4-aminobenzyl alcohol were purchased from Aladdin Biochemical Technology Co., Ltd. (Shanghai, China). Nitric acid, N,N-dimethylformamide (DMF), and NaOH were purchased from Sinopharm chemical Reagent Co., Ltd. (Shanghai, China). Polyvinylpyrrolidone (PVP, K22-K27, average molecular weight = 40,000) was purchased from Sun Chemical Technology Co., Ltd. (Shanghai, China). All reagents were used without further purification. Water was Milli-pore grade in all the experiments.

### 3.2. Characterization

JEOL-7800F SEM (JEOL, Tokyo, Japan) and Talos F200X TEM (Thermo Fisher Scientific, Waltham, MA, USA) were used to image the morphology of the samples. Rigaku D/max-2550 VB XRD (Rigaku, Tokyo, Japan) with a Cu Kα radiation source was used to characterize the crystal structure of the samples. XPS characterization was performed using an ESCALAB 250 Xi spectrometer (Thermo Fisher Scientific, Waltham, MA, USA). The optical absorption spectrum was measured using a UV-3600i Plus UV−Visible spectrometer (Shimazu, Tokyo, Japan). All characterizations using above instruments were performed in Characterization Center and our own lab at Jiangsu University in Zhenjiang, China.

### 3.3. Acid Pretreatment of Carbon Nanotubes

To 120 mL of nitric acid solution, we added 2 g of CNTs and dispersed them evenly by ultrasonication. This suspension was then transferred into a three-necked flask and refluxed at 120 °C for 4 h. The CNTs were separated and washed with deionized water until they were neutral. Finally, the treated CNTs were dried in a vacuum oven at 60 °C overnight.

### 3.4. Synthesis of Bi_2_Te_3_/CNT Nanohybrid

Initially, 0.4 g of PVP was dissolved in 36 mL of ethylene glycol. Subsequently, treated CNTs, 0.4660 g of Bi_2_O_3_, and 0.4792 g of TeO_2_ powders were added to this solution. To this solution, 4 mL of 5 M NaOH solution was added and vigorously stirred for 30 min. The resulting suspension was sealed in a 100 mL Teflon-lined steel autoclave and heated at 210 °C for 24 h [30,31]. The synthesized products were collected by centrifugation, washed with distilled water and absolute ethanol, and finally dried at 60 °C for 12 h. During the synthesis, the content of CNTs was set to be 0%, 10%, 30%, 50%, and 70% of the mass of Bi_2_Te_3_. The obtained samples were Bi_2_Te_3_, Bi_2_Te_3_/10%CNT, Bi_2_Te_3_/30%CNT, Bi_2_Te_3_/50%CNT, and Bi_2_Te_3_/70%CNT.

### 3.5. Catalytic Generation of H_2_O_2_

Typically, 60 mg of the sample was dispersed in 50 mL water in a 100 mL beaker under sonication. Then, this solution was purged with oxygen for 30 min. A cooling coil tube and magneton were placed in the beaker to cool and stir the solution. This system was then placed in an oil bath set at various temperatures, and the cooling coil tube was connected with a cycling chilling machine with cooling water temperature set at 5 °C. The ΔT is the temperature difference between the oil bath and cooling coil. The ΔT is tunable by simply varying the oil bath temperature.

### 3.6. Determination of Hydrogen Peroxide Concentration

Firstly, two solutions A and B were prepared [32]. Solution A was composed of 0.4 M KI, 0.06 M NaOH, and 0.1 mM ammonium molybdate. Solution B was 0.1 M Potassium Hydrogen Phthalate. During catalysis, 1 mL liquid was extracted from the reaction solution and mixed with 0.5 mL of A and 0.5 mL B. The mixed solution was tested by UV-Vis spectrophotometer to monitor the absorption peak at 351 nm.

### 3.7. Electrochemical Measurements

The TE current was tested using a standard three-electrode electrochemical cell of the CHI760e electrochemical workstation. Cooper-type regimens coated with the Bi_2_Te_3_/CNT catalysts, Ag/AgCl and platinum net (10 × 10 × 1 mm) were used as the working electrode, reference electrode, and counter electrode, respectively. Deoxygenated Na_2_SO_4_ solution (0.1 M) was used as the electrolyte [33]. The electrodes were placed in a beaker filled with the electrolyte. This cell was immersed in an oil bath by installing a cooling coil connected to a cycling chilling machine with the cooling water temperature set at 5 °C, similar to the setup for the H_2_O_2_ generation. During the test, the chilling machine and the oil bath pot worked at the same time to create a ΔT. The TE current and EIS of the different temperature differences was measured.

## 4. Conclusions

In summary, we have demonstrated a unique Bi_2_Te_3_/CNT nanohybrid for TECatal H_2_O_2_ production, utilizing waste heat to drive the synthesis. The temperature gradient induces the TE effect in Bi_2_Te_3_, promoting the separation of charge carriers for H_2_O_2_ generation. The CNTs hybridized on the Bi_2_Te_3_ surface and improved the acceptance and transport of TE charges, leading to enhanced electron–hole pair separation. This work presents an innovative approach for synthesizing important chemicals using low-grade waste thermal energy.

## Data Availability

The data presented in this study are available on request from the corresponding author.
